# Community Water Fluoridation in Focus: A Comprehensive Look at Fluoridation Levels across America

**DOI:** 10.3390/ijerph20237100

**Published:** 2023-11-23

**Authors:** Man Hung, Amir Mohajeri, Jody Chiang, Jungweon Park, Beatrice Bautista, Chase Hardy, Martin S. Lipsky

**Affiliations:** 1 College of Dental Medicine, Roseman University of Health Sciences, South Jordan, UT 84095, USA; 2 Department of Orthopaedics, University of Utah, Salt Lake City, UT 84108, USA; 3 College of Social Work, University of Utah, Salt Lake City, UT 84112, USA; 4 Department of Veterans Affairs Medical Center, Salt Lake City, UT 84148, USA; 5 College of Dentistry, Ohio State University, Columbus, OH 43210, USA; 6 College of Dentistry, University of Texas Health Sciences, San Antonio, TX 78253, USA; 7 Institute on Aging, Portland State University, Portland, OR 97201, USA

**Keywords:** fluoride, fluoridated water, fluoridation, fluorination, public health dentistry, caries prevention

## Abstract

*Objective*: This study reports on the number and percentage of community water systems (CWSs) meeting fluoride concentration standards set by the U.S. Department of Health and Human Services (DHHS). The study also explored changes in the population exposed to optimally fluoridated water in these systems between 2006 and 2020. *Methods*: This study analyzed U.S. Centers for Disease Control and Prevention data from 2006 to 2020, tabulating state-specific CWS fluoridation rates, ranking them, and calculating the percent change. *Results*: In 2020, 72.7% of the US population received CWS water, with 62.9% of those individuals served by a CWS system meeting DHHS fluoridation standards. This compares to 69.2% receiving CWS water in 2006 and 74.6% in 2012. The overall change in those receiving fluoridated water was 1.4%, from 61.5% in 2006 to 62.9% in 2020. State-specific percentages ranged from 8.5% in Hawaii to 100% in Washington DC in 2020 (median: 76.4%). *Conclusions*: Although endorsed by the American Dental Association, the percentage of individuals receiving fluoridated water did not increase substantially from 2006 to 2020, indicating that there has not been much progress toward meeting the Healthy People 2030 goal that 77.1% of Americans receive water with enough fluoride to prevent tooth decay.

## 1. Introduction

Community water fluoridation (CWF) is the practice of adjusting the level of fluoride in public water supplies to a concentration optimal for the prevention of tooth decay [1]. This public health initiative is backed by numerous studies that underscore its efficacy; even amid widespread use of fluoride-containing dental products, such as toothpastes and mouthwashes, CWF contributes an additional reduction in dental decay by over 25%, which translates to significant cost savings in dental health expenditures, with estimates placing the per capita savings at over thirty dollars [2,3]. The robust evidence supporting the benefits of fluoridation has garnered endorsements from a spectrum of authoritative health organizations. These endorsements come from the American Dental Association, the American Academy of Pediatrics, the U.S. Public Health Service, and international bodies like the World Health Organization and the Centers for Disease Control and Prevention (CDC) [4]. The collective support from these organizations stems from a recognition of the substantial impact CWF has had on oral health. Its effectiveness in reducing dental decay ranks CWF among the most important public health achievements of the 20th century [5].

Despite the widespread endorsement, skepticism about the safety and benefits of fluoridation persists. Several studies report on fluoride’s negative effect on cognition, especially during fetal development and among children [6,7,8]. These studies propose that excess fluoride crosses the blood–brain barrier, causing structural and cognitive alterations in the central nervous system, or during pregnancy when fluoride can cross the placenta, and affect fetal development [9,10,11,12,13]. However, a recent systematic review challenged the quality of these studies and found insufficient evidence to conclude that fluoride is associated with neurological damage [14]. A meta-analysis by Kumar and colleagues also determined that water fluoridation at the concentration used in community water fluoridation is not associated with lower IQ scores in children [15].

In 2015, the U.S. Department of Health and Human Services (DHHS) reduced the recommended fluoride level for community water systems (CWSs) from a range of 0.7–1.2 mg fluoride ion/L to 0.7 mg fluoride ion/L. This change sought to balance maintaining dental caries prevention while minimizing the risk of fluorosis and other potential health harms [16]. Dental fluorosis is defined as changes in the appearance of tooth enamel caused by overexposure to fluoride during enamel formation, and varies from small white spots or pits on the enamel to distinct brown stains in severe cases [17,18]. Its risk increases as children ingest higher levels of fluoride [17]. Those opposing fluoridation also assert that CWF increases the risk of joint problems, heart disease, kidney disease, and cancer [19]. Carstairs notes, that perhaps fluoride proponents were too hasty in declaring that community water fluoridation was the best or only solution for dental decay [20]. Many other countries opt not to add fluoride to water and around 5 to 6% of the global population receive water fluoridated at the recommended level, with nearly half of them living in the United States [21].

In 2016, 72.8% of those who were served by CWSs received optimally fluoridated water [22], which fell 4.2% below the Healthy People (HP) 2030 objective that 77.1% of Americans served by CWS should receive optimally fluoridated water. Even though the American Public Health Association comprehensively reviewed potential harms and endorsed CWF, some public doubt about the safety and value of CWF persists [23]. Compounding this opposition is the belief that government policies such as fluoridation interfere with personal choice and freedom [24]. The impact of anti-fluoridation concerns over time remains uncertain.

This study reports on the prevalence and distribution of CWF within CWS. It examines both the number and proportion of CWS that have adhered to recommended fluoride levels over the period from 2006 to 2020. The findings are particularly relevant for public health agencies striving to monitor and enhance the reach of water fluoridation programs. The results also hold significant weight for policy formulation, as they provide an empirical basis to assess the strides made towards the target set by Healthy People 2030—a national objective that aims for 77.1% of the population to benefit from fluoridated community water by the end of the decade.

## 2. Methods

### 2.1. Data Source

This study used data collected by the CDC in the US from 2006 to 2020 to monitor the fluoridation status of approximately 54,000 CWSs [25]. The Water Fluoridation Reporting System (WFRS) is the principal tool the CDC uses to aid states in monitoring the quality of their water fluoridation programs. The WFRS collects information from state drinking water programs and the CDC uses the data to compile water fluoridation statistics. Each year, the CDC collaborates with state programs to improve the accuracy of CWS statistics by finding and addressing inconsistencies between the US Environmental Protection Agency’s Safe Drinking Water Information System and WFRS databases [26]. Every two years, the CDC publishes a National Water Fluoridation Statistics report, which estimates the proportion of the US population receiving fluoridated water and the percentage of the CWS population receiving fluoridated water in each state. Further details regarding the methodology for calculating fluoridation statistics can be found at https://www.cdc.gov/fluoridation/statistics/index.htm (accessed on 1 November 2023).

### 2.2. Mesures

Measures captured using the WFRS and reported by the CDC include the total number of CWSs in the US, the number of CWSs providing fluoridated water, the number of CWSs adjusting fluoride levels, the number of CWSs consecutive to systems with optimal fluoride levels, the number of CWSs with naturally occurring fluoride at or above optimal levels, the population served by CWSs with naturally occurring fluoride at or above optimal levels, the percentage of the US population on CWSs receiving fluoridated water, the percentage of the US population receiving fluoridated water, the total US population on fluoridated drinking water systems, the US population on CWSs, and the total US population. A CWS is defined as a water system that provides a year-round supply of water to the same population of at least 25 persons in their primary residences or at least 15 primary residences [27]. A consecutive system is defined as a water system that purchases water from another system and does not adjust the fluoride concentration. If a consecutive water system purchases non-fluoridated water and then adjusts fluoride to optimal levels, it is considered adjusted in WFRS [28].

State populations served by CWS reported in WFRS are estimated by using a product of the US Census state population estimate and the US Geological Survey’s estimate of the percentages of state populations on public water systems. The CDC uses the following steps to determine the fluoridated population in each state: for the Adjusted State CWS Population, multiply the Census Bureau State Population Estimate using the US Geological Survey estimate for the Percentage of State Population served by Public Supply; for a “Control Factor”, divide the Adjusted State CWS Population by the state-reported Population Served by CWSs from WFRS. Each water system’s reported population is multiplied by the Control Factor to create Individually Adjusted CWS Populations. Finally, the State calculates the Fluoridated Population by adding all the Individually Adjusted CWS populations for the fluoridated systems in a state. The fluoridated population divided by the population served by CWSs yields the percentage of the US population on CWSs receiving fluoridated water.

### 2.3. Data Analyses

CWS fluoridation data were tabulated for each state and ranked by fluoridation percentage. For each state, the percentage difference between 2006 and 2020 was calculated. Calculations assessed the intervals from 2006 to 2012 (before the revised fluoridation standard of 2015), and from 2016 to 2020 (which represents the period after the 2015 fluoride standard change). The study used descriptive statistics to examine the fluoridation measures from the years 2006 to 2020 on the population level. Additionally, graphical representations were also plotted to display the percentage of the US population receiving fluoridated water, facilitating a clearer understanding of the temporal progression of CWS fluoridation.

## 3. Results

**Figure 1:**
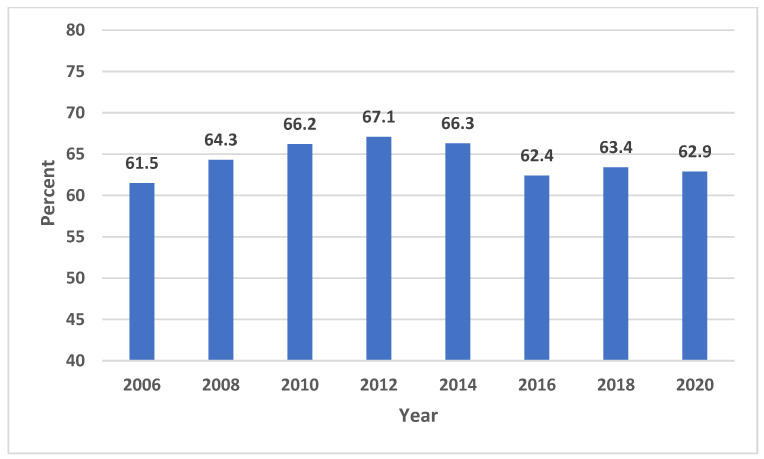
Percentage of United States population receiving fluoridated water.

In 2020, CWSs supplied water to over 287 million individuals in the US, and 72.7% of those individuals received CWS-supplied fluoridated water. In comparison, the percentage of the US population who received water from CWSs was 69.2% in 2006, and 74.6% in 2012 (Table 1). Among the total US population in 2020, 62.9% received water that met fluoridation standards. The percentage of those receiving fluoridated water ranged from a low of 61.5% in 2006 to a high of 67.1% in 2012 (Figure 1).

Table 1 displays water fluoridation supply statistics in two-year intervals. From 2006 to 2020, the number of Americans on CWSs increased from about 262 million to 287 million persons. Table 1 also shows the trends in populations supplied by CWSs with naturally occurring fluoride at or above recommended levels. Approximately 8 million persons received water with sufficient naturally occurring fluoride concentrations at or above optimal levels in 2006, versus 12 million persons in 2020. 

**Figure 2:**
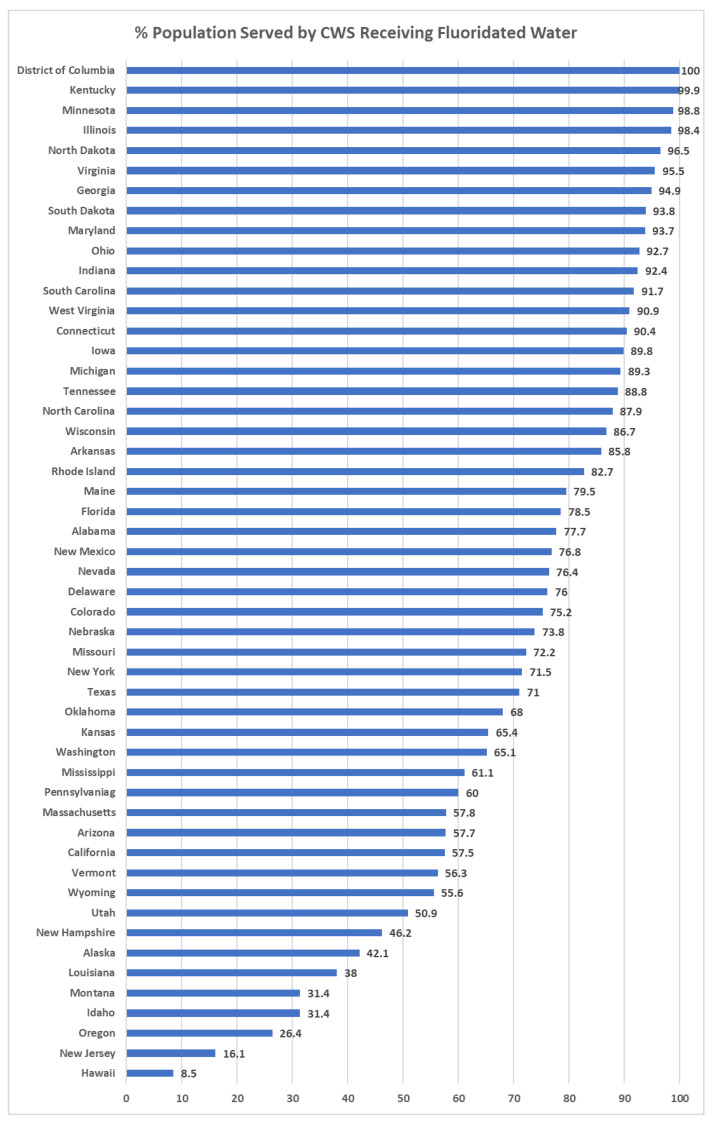
Percent of the United States population served by community water systems receiving fluoridated water across states.

Although there was an absolute increase of 25 million people receiving fluoridated water, the US population increased by over 32 million during the same period, so the difference in the proportion between those receiving fluoridated water in 2006 (61.5%) and in 2020 (62.9%) was quite small, at 1.4%. Table 2 shows the percentage of the population meeting fluoridation standards by state in 2006, 2012, and 2020. State-specific percentages in 2020 ranged from 8.5% in Hawaii to 100% in Washington DC (median: 76.4%). Washington DC exhibited the greatest percentage of people receiving CWS fluoridated water, followed by the states of Kentucky, Minnesota, and Illinois (Figure 2).

Table 2 displays the trends of fluoridation over separate time intervals, 2006 to 2012 and 2016 to 2020. Between 2006 and 2012, 26 states reported increases in the percentage of their populations on CWSs receiving fluoridated water, ranging from 0.1% in Kentucky and Minnesota to 36.6% in California (median: 1.6%), while 23 states had decreases, ranging from 0.1 to 8.0% (median: 1.2%). Between 2016 and 2020, 22 states reported a rise in the percentage of their population supplied by fluoridated CWSs, with percentage-point increases that ranged from 0.1% in Mississippi and Vermont to 5.4% in Pennsylvania (median: 0.5%). In 26 states, there were decreases, ranging from 0.1% in Arizona, Illinois, and Massachusetts to 11.4% in Delaware (median: 1.4%) (Table 2).

Despite a decrease in the number of CWSs that provide fluoridated water from 18,030 in 2016 to 17,558 in 2020, there was little difference between the number of individuals receiving CWS fluoridated water, which represents the reporting years after the DHHS fluoride standard modification in 2015. From 2006 to 2020, the number of CWSs adjusting for fluoride level decreased from 6368 in 2006 to 5728 in 2020.

## 4. Discussion

Since 1990, the DDHS has used the percentage of the population on CWSs receiving fluoridated water to set national health goals [29]. In 2020, CWSs that met DHHS fluoride concentration standards served over 209 million individuals, or 72.7% of the population supplied by CWSs. Among the total US population in 2020, 62.9% received water that met fluoridation standards. Because of its oral health benefits, HP 2030 set a national goal that 77.1% of Americans served by CWSs receive water with enough fluoride to prevent tooth decay [22]. Several key groups and public agencies endorse fluoridation, and one might expect that these recommendations and endorsements would lead to an increase in the percentage of individuals receiving fluoridated water. However, this study found that neither the percentage of the US population receiving fluoridated water nor the percentage of the US population on CWSs receiving fluoridated water increased significantly from 2006 to 2020. Over the study period, the percentage of those receiving fluoridated water peaked in 2012, but then decreased from 2012 to 2020, despite the HP goal to increase fluoridation. 

Several reasons may explain the lack of significant progress toward meeting the HP 2030 target for fluoridation. One is that evidence associates adverse health effects such as dental and skeletal fluorosis with chronic overexposure to fluoride, although these events are linked to levels above the DHHS standard and dental fluorosis only occurs during the tooth mineralization stage. Chronic exposure after tooth eruption does not cause dental fluorosis. While Kumar, Miranda, and others found little evidence linking recommended levels of fluoride supplementation to neurotoxicity, recent systematic reviews suggest that neurotoxicity may occur at fluoride concentrations ranges previously considered safe, such as 0.7–1.2 mg/L [14,15,30]. Grandjean also reviewed the literature and expressed concern that lower levels of fluoride intake during early development may cause IQ deficits and concluded that 0.30 mg/L is the approximate threshold for fluoride neurotoxicity at developmental age [13], even though a 2020 Canadian panel found insufficient evidence to conclusively conclude that exposure to water fluoride levels at 0.7 mg/L affect neurological development [31]. In addition, groups such as the Fluoride Action Network, which oppose fluoridation, have gained traction with the public [32]. They believe and promote the idea that fluoride toxicity has been overlooked and underestimated, its benefits overestimated, and that ending water fluoridation protects public health. Others view fluoridation as a violation of civil liberties and their constitutional right to freedom of choice [22]. One consequence of these arguments is that cities such as Portland, Oregon, and Juneau, Alaska voted either to not initiate fluoridation or to stop adding fluoride to their water systems [33,34]. Our finding that the percentage of individuals receiving fluoridated water did not significantly increase from 2006 to 2020 highlights the influence of these factors. 

Fluoridation critics also contend that the wide availability of fluoride-containing products, such as toothpaste and mouth rinses, preclude the need for fluoridation. However, drinking water still accounts for 40 to 70% of total fluoride intake in children, and about 60% of total fluoride intake in adults, underscoring the need for fluoridation [16]. States that rely on fluoride sources other than water, such as Hawaii, where less than 9% of the population receives fluoridated water, experience some of the poorest oral health outcomes. Hawaiian children suffer from dental caries at almost twice the average rates of those reported for mainland children [35]. 

While it may be politically, logistically, and financially challenging to fluoridate the remaining CWSs, it is important to recognize that because of a favorable cost-to-benefit ratio, even small increases in fluoridation can yield substantial benefits. A systematic review found that per capita annual costs ranging from $0.11 to $24.38 yielded benefits from $5.49 to $93.19 [36]. A 2013 study estimated that community water fluoridation saved an average of $32.19 per person, and a single percentage point increase nationally in fluoridation could save over 100 million dollars annually [37]. Similarly, a growing body of evidence demonstrates that decreases in fluoridation adversely affect oral health and increase costs [34]. For example, a Canadian study comparing the cities of Calgary (fluoridation stopped in 2011) and Edmonton (still fluoridated) found a significantly higher prevalence of caries in the primary dentition in Calgary than in Edmonton [38]. 

Another key finding was that while the percentage of those receiving fluoridated water did not increase, the number of CWSs with naturally occurring fluoride at or above recommended levels increased from 3339 in the year 2006 to 5626 in the year 2020. Although this may reflect the 2015 change in the fluoride standard, one concern is that an increased number of individuals exposed to natural fluoride may experience fluoride levels that could increase fluorosis prevalence [39]. While most water sources contain safe levels of fluoride, individuals living in mountainous regions, or in areas where agricultural and industrial waste may contaminate water, could be exposed to high and unsafe fluoride levels.

Failure to make significant progress towards the HP 2030 goal raises a crucial question: why does the percentage of the population with access to optimally fluoridated water remain below expectations when the DHHS set guidelines for water fluoridation levels that show no strong evidence of adverse health outcomes? Addressing this question necessitates a multifaceted approach. First, there is a need for more research to investigate potential barriers to adoption. This includes examining public perceptions, understanding the dissemination of misinformation, evaluating infrastructure limitations, and assessing financial and policy constraints. Furthermore, the communication strategies surrounding the promotion of fluoridated water may require refinement. Lessons from the COVID-19 pandemic and vaccine opposition highlight that support for public health measures constitutes a serious challenge and illustrates how poor communication can undermine public trust [40]. Effective messaging is vital to ensure public support for fluoridation initiatives. This may involve targeted educational programs that address common misconceptions and highlight the safety and benefits of fluoride in preventing dental disease. 

### Limitations

This study has several limitations. The data are from 2006 to 2020, and more recent data may show different trends. Global events such as the COVID-19 pandemic and political movements to support individual rights regarding vaccination may have also affected fluoride policies. Participation in the WFRS is voluntary and some state fluctuations may reflect changes in data reporting rather than true gains or losses in fluoridated water systems.

Another possible limitation is that the CDC modified its collection methodology in 2016. However, Hamilton et al. found that the change minimally affected the percentage of the total CWS population receiving fluoridated water [41]. Studies showing increases in the prevalence of fluorosis may also have impacted policy [17]. The DHHS recommendation to lower fluoride levels midway through the study period might be a confounding factor. However, this would likely skew results to an increased number of individuals receiving fluoridated water at recommended levels. Additionally, plasma fluoride levels more accurately reflect biological activity, and this study reported only on water fluoride levels. Finally, the data on who receives fluoridated water do not distinguish those who might drink bottled water or use filters to remove fluoride.

## 5. Conclusions

By examining the changes in the population that have had access to optimally fluoride from 2006 to 2020, this study offers insights into how the dynamics of public health interventions may shape policy. In 2020, 72.7% of the US population was supplied by CWS, and 62.9% of the total population received water that met DHHS fluoridation standards. Although public health agencies, the American Dental Association, and other healthcare groups advocate for fluoridation as a safe, simple, and cost-effective public health measure, the percentage of individuals receiving fluoridated water has not increased significantly since 2006, indicating little progress toward achieving the 77.1% goal of Healthy People 2030. 

## Figures and Tables

**Table 1:** Water fluoridation statistics by year provided by the Centers for Disease Control and Prevention.

Measures	2006	2008	2010	2012	2014	2016	2018	2020
Number of CWSs adjusting fluoride	6368	6143	6042	5999	5919	5817	5753	5728
Number of CWSs consecutive to systems with optimal fluoride levels	6705	6176	6795	6342	6015	6230	6340	5696
Number of CWSs providing fluoridated water	16,412	16,977	18,427	18,502	18,186	18,030	17,917	17,558
Number of CWSs with naturally occurring fluoride at or above optimal levels	3339	4658	5590	6151	6205	5865	5727	5636
Percentage of U.S. population on CWSs receiving fluoridated water	69.2	72.4	73.9	74.6	74.4	72.8	73.0	72.7
Percentage of U.S. population receiving fluoridated water	61.5	64.3	66.2	67.1	66.3	62.4	63.4	62.9
Population served by CWSs with naturally occurring fluoride at or above optimal levels	8,078,890	8,805,304	10,077,922	11,116,202	11,883,007	11,283,910	12,059,342	11,578,079
Total number of CWSs in the United States	53,429	55,396	54,293	52,734	*	52,286	52,211	51,373
Total U.S. population on fluoridated drinking water systems, persons	184,028,038	195,545,109	204,283,554	210,655,401	211,393,167	201,565,162	207,426,536	209,145,650
Total U.S. population, persons	299,398,484	304,059,724	308,745,538	313,914,040	318,857,056	323,127,513	327,167,434	331,501,080
U.S. population on community water systems, persons	262,690,043	269,911,707	276,607,387	282,534,910	284,099,832	276,969,134	284,075,868	287,798,584

* Lack of data from original source file provided by the Centers for Disease Control and Prevention.

**Table 2:** Percentage of population served by community water systems who received fluoridated water in 2006, 2012, and 2018, and percentage changes over time by state.

State	2006 %	2012 %	2016 %	2020 %	% Difference (2006–2012)	% Difference (2016–2020)
United States	69.2	74.6	72.8	72.7	5.4	−0.1
Alabama	82.9	78.6	78.0	77.7	−4.3	−0.3
Alaska	59.5	52.9	49.6	42.1	−6.6	−7.5
Arizona	56.1	57.8	57.8	57.7	1.7	−0.1
Arkansas	64.4	66.9	85.6	85.8	2.5	0.2
California	27.1	63.7	60.6	57.5	36.6	−3.1
Colorado	73.6	72.4	74.9	75.2	−1.2	0.3
Connecticut	88.9	90.3	89.5	90.4	1.4	0.9
Delaware	73.6	86.3	87.4	76	12.7	−11.4
District of Columbia	100	100	100.0	100	0	0.0
Florida	77.7	78.0	77.0	78.5	0.3	1.5
Georgia	95.8	96.3	96.2	94.9	0.5	−1.3
Hawaii	8.4	10.8	11.3	8.5	2.4	−2.8
Idaho	31.3	36.1	32.2	31.4	4.8	−0.8
Illinois	98.9	98.5	98.5	98.4	−0.4	−0.1
Indiana	95.1	94.8	94.3	92.4	−0.3	−1.9
Iowa	92.4	92.0	90.3	89.8	−0.4	−0.5
Kansas	65.1	63.6	66.4	65.4	−1.5	−1.0
Kentucky	99.8	99.9	99.9	99.9	0.1	0.0
Louisiana	40.4	43.4	44.2	38	3.0	−6.2
Maine	79.6	79.4	79.3	79.5	−0.2	0.2
Maryland	93.8	97.2	93.4	93.7	3.4	0.3
Massachusetts	59.1	70.4	57.9	57.8	11.3	−0.1
Michigan	90.9	90.2	89.7	89.3	−0.7	−0.4
Minnesota	98.7	98.8	98.8	98.8	0.1	0.0
Mississippi	50.9	58.2	61.0	61.1	7.3	0.1
Missouri	79.7	76.4	76.8	72.2	−3.3	−4.6
Montana	31.3	32.0	33.7	31.4	0.7	−2.3
Nebraska	69.8	71.2	71.6	73.8	1.4	2.2
Nevada	72.0	73.5	75.0	76.4	1.5	1.4
New Hampshire	42.6	46.0	46.5	46.2	3.4	−0.3
New Jersey	22.6	14.6	14.6	16.1	−8.0	1.5
New Mexico	77.0	77.0	77.0	76.8	0.0	−0.2
New York	72.9	71.8	71.7	71.5	−1.1	−0.2
North Carolina	87.6	87.5	87.7	87.9	−0.1	0.2
North Dakota	96.2	96.7	95.8	96.5	0.5	0.7
Ohio	89.3	92.2	92.5	92.7	2.9	0.2
Oklahoma	73.5	70.1	69.6	68	−3.4	−1.6
Oregon	27.4	22.6	22.6	26.4	−4.8	3.8
Pennsylvania	54.0	54.6	54.6	60	0.6	5.4
Rhode Island	84.6	83.9	84.5	82.7	−0.7	−1.8
South Carolina	94.6	93.8	91.4	91.7	−0.8	0.3
South Dakota	95.0	93.6	93.6	93.8	−1.4	0.2
Tennessee	93.7	89.7	88.4	88.8	−4.0	0.4
Texas	78.1	79.6	67.6	71	1.5	3.4
Utah	54.3	51.7	52.7	50.9	−2.6	−1.8
Vermont	58.7	56.1	56.2	56.3	−2.6	0.1
Virginia	95.0	96.0	96.3	95.5	1.0	−0.8
Washington	62.9	63.6	63.9	65.1	0.7	1.2
West Virginia	91.7	91.1	90.3	90.9	−0.6	0.6
Wisconsin	89.7	89.4	88.3	86.7	−0.3	−1.6
Wyoming	36.4	43.6	57.1	55.6	7.2	−1.5

## Data Availability

Data are available at https://www.cdc.gov/fluoridation/statistics/index.htm (accessed on 1 November 2023).
